# Bottom up approach of metal assisted electrochemical exfoliation of boron towards borophene

**DOI:** 10.1038/s41598-022-20130-w

**Published:** 2022-09-20

**Authors:** Krzysztof Sielicki, Klaudia Maślana, Xuecheng Chen, Ewa Mijowska

**Affiliations:** Faculty of Chemical Technology and Engineering, Nanomaterials Physicochemistry Department, West Pomeranian University of Technology, Piastow Ave. 42, 71-065 Szczecin, Poland

**Keywords:** Chemistry, Materials science, Nanoscience and technology

## Abstract

Electrochemical exfoliation of nonconductive boron to few-layered borophene is reported. This unique effect is achieved via the incorporation of bulk boron into metal mesh inducing electrical conductivity and opening a venue for borophene fabrication via this feasible strategy. The experiments were conducted in various electrolytes providing a powerful tool to fabricate borophene flakes with a thickness of ~ 3–6 nm with different phases. The mechanism of electrochemical exfoliation of boron is also revealed and discussed. Therefore, the proposed methodology can serve as a new tool for bulk scale fabrication of few-layered borophene and speed up the development of borophene-related research and its potential application.

## Introduction

Two-dimensional (2D) materials have caught huge interest in recent years due to their unique properties like electrical conductivity or prominent active surface. The development of graphene materials put other 2D materials into the spotlight, therefore new 2D materials are widely explored. Notwithstanding well-known graphene, transition metal dichalcogenide (TMD) like WS_2_^1^, MoS_2_^2^, MoSe^3^, and WSe^4^ have been intensively studied lately. Despite the above-mentioned materials, hexagonal boron nitride (hBN), black phosphorus and recently successfully manufactured borophene. Among them, borophene as one of the youngest 2D systems has focused huge attention. It is layered as graphene, however, it presents intriguing properties due to its anisotropy, polymorphism and crystal structure. Bulk boron occurs in a B_12_ icosahedron as a basic structural unit but the various types of boron crystals are formed through different connections and bonding methods in the B_12_. Therefore, the boron block is not typically layered like graphene or graphite and this leads to more complex methods to fabricate borophene. Additionally, many polymorphism forms of borophene e.g. *α*, *β*, *α1*, *pmmm* make it even more complicated^5^. Different phases achieved during synthesis directly impact the properties of borophene. Consequently, the development of synthesis methods that can lead to the fabrication of particular phases of borophene with a big lateral size of flakes and its low thickness should be currently deeply explored.

Numerous synthesis methods of 2D materials are based on sonochemical processes, where bulk material is put into a solvent, usually organic, and subjected to ultrasounds for many hours. Ranjan et al.^6^ successfully exfoliated the bulk boron to borophene with the above-mentioned method. They investigated many organic solvents (methanol, ethanol, isopropanol, acetone, DMF, DMSO) and proved that exfoliation with the assistance of the ultrasounds is a facile method to fabricate large and thin flakes of borophene. Furthermore, they proved that the modified Hummers method can be applied to exfoliate boron as well. Liquid exfoliation was proved by others: Lin et al.^7^ utilized crystalline boron as a source to synthesize few-layer β_12_ borophene sheets and further utilized it in borophene-based Li–S batteries, Li et al.^8^ proved that few-layered borophene flakes can be achieved via sonochemical synthesis and utilized as supercapacitor electrodes. Despite that, atomic layer deposition (ALD) is one of the bottom-up methods to synthesize the borophene as well. Mannix et al.^9^ deposited boron atoms onto atomically clean silver support. This approach yields ultrapure borophene sheets; however, it is strongly limited to the lab-scale production of borophene due to strict conditions of the process—ultra-high vacuum. Therefore, new efficient strategies for borophene fabrication have to be developed with the mechanism of growth/exfoliation explained followed by a precise theoretical analysis of its properties such as polymorphism, and electrical and thermal transport. The mechanism of borophene growth on Cu(111) substrate was discussed and explained by H. Liu et al.^10^. It was proved that the tendency of boron atoms to form 2D compact clusters based on triangular units and the steady decline in formation energy with increasing cluster size suggests that a 2D boron cluster on a Cu substrate may continue to grow indefinitely. A more detailed analysis of 2D boron sheets was presented by D. Li et al.^11^, where various substrates were described and potential application was discussed. It is clearly stated that there are some differences between theoretical calculations and experimental results. Therefore, theoretical calculations are required to fully understand the borophene properties and growth mechanism. One of the methods to achieve that is exfoliation of boron by utilization of simple adhesive tape but this is still too small scale to explore fundamental properties and revise its practical applications^12^.

One of the promising routes to design exfoliated 2D materials from their bulk counterparts is electrochemical exfoliation. Here, one of the electrodes is composed of bulk material. Typically, compounds that are usually exfoliated via electrochemical methods possess high electrical conductivity. They are used in the form of compressed rods or tablets. Graphite can be successfully exfoliated in that manner^13^ due to its high electric conductivity. Achee and his team^14^ have successfully exfoliated graphite by changing the graphite rode into compressed graphite in the presence of a membrane used to avoid the disintegration of bulk material. Similarly, other bulk layered materials were successfully exfoliated, e.g. via Janus electrochemical exfoliation^15^. Likewise, layered black phosphorus was electrochemically exfoliated where ions in acid electrolyte diffused into the spaces between layers due to applied voltage^16^. Unfortunately, the same methods can not be simply applied to exfoliate boron to borophene due to the low conductivity of bulk material. However, what would happen when bulk boron powder would be incorporated into metal meshes (nickel-Ni or copper-Cu) to serve as an electrode? Is it possible to induce the conductivity to boron which could be further electrochemically exfoliated as conducting bulk layered systems? What about phases of the designed few-layered borophene?

**Figure 1:**
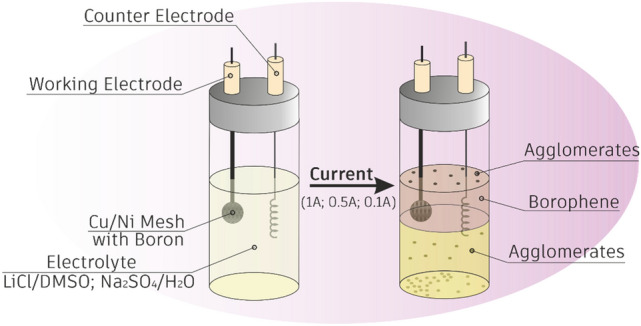
Schematic representation of the electrochemical exfoliation of boron.

In this study, we answered these questions proving that this facile strategy delivered a new universal approach toward the fabrication of few-layered borophene what is schematically presented in Fig. 1.

## Materials and methods

### Materials

Lithium chloride (LiCl, 99.0%, CAS:7447–41-8) and Boron powder (B, CAS: 7440–42-8) were purchased from Sigma Aldrich (USA). Sodium sulfate (Na_2_SO_4_, ≥ 99.0%, CAS: 7757–82-6) was delivered from Chempur (Poland). Dimethyl sulfoxide (DMSO, CAS: 67–68-5) from Karpinex company (Poland) were used.

### Structure characterization

Atomic Force Microscopy (AFM MultiMode 8 (Bruker)) provide information about the thickness and lattice size of exfoliated materials. High-resolution transmission electron microscopy (HR-TEM) imaging was performed with FEI Tecnai F20 microscope at an accelerating voltage of 200 kV. Atomic absorbance spectroscopy (AAS) analysis was performed using Hitachi Polarized Zeeman Atomic Absorption Spectrophotometers Flame atomizers to determine the migration of metal ions into the solution during the electrochemical process of exfoliation. The zeta-potential of bulk boron was measured and performed on a Zeta Sizer (ZS Nano ZEN 3600, Malvern) to determine the surface potential of bulk boron. The chemical composition and relative atomic percentages on the surface of the samples were studied by X-ray Photoelectron Spectroscopy (XPS). The measurements were conducted using Mg Ka (hν = 1253.6 eV) radiation in a PREVAC (Poland) system equipped with a Scienta SES 2002 (Sweden) electron energy analyzer operating with constant transmission energy (Ep = 50 eV). The analysis chamber was evacuated to a pressure below 5 × 10^−9^ mbar.

### Electrochemical exfoliation

Typically, bulk boron powder 0.1 g was first pressed into discs made of metal mesh (nickel or copper) using a hydraulic press. The diameter of the disc was 15 mm. As prepared disks were used as electrodes. Two types of electrolytes were used: (i) 1 M solution of LiCl in DMSO and (ii) 1 M solution of Na_2_SO_4_ in DI water. Platinum wire served as the auxiliary electrode. A schematic representation of work station is shown in Fig. 1*.* During the process of electrochemical exfoliation specified electric current (1 A, 0.5 A or 0.1 A) was applied between the cathode and anode. The time of each experiment was 1 h. After the process the supernatant was collected, centrifuged at the speed of 5000 rpm and washed several times (3–5) with DI water.

## Results

Different parameters such as time and the distance between electrodes have an impact on the morphology of the final product of electrochemical exfoliation. Here, we investigate the influence of electrolyte, applied current (1 A, 0.5 A and 0.1 A; voltage 30 V) and sort of metal mesh (Ni versus Cu) and examine their effect on the borophene sheets fabrication in terms of thickness and flake size. Two different electrolytes have been tested: (i) 1 M lithium chloride (LiCl) in dimethyl sulfoxide (DMSO) and (ii) 1 M sodium sulfate (Na_2_SO_4_) in deionized (DI) water. In the first one, during the process lithium cations (Li^+^) will intercalate the boron connected to the negative charge. In the latter case sulfate anions (SO_4_^2−^) will intercalate the boron supplied with a positive charge.

**Figure 2:**
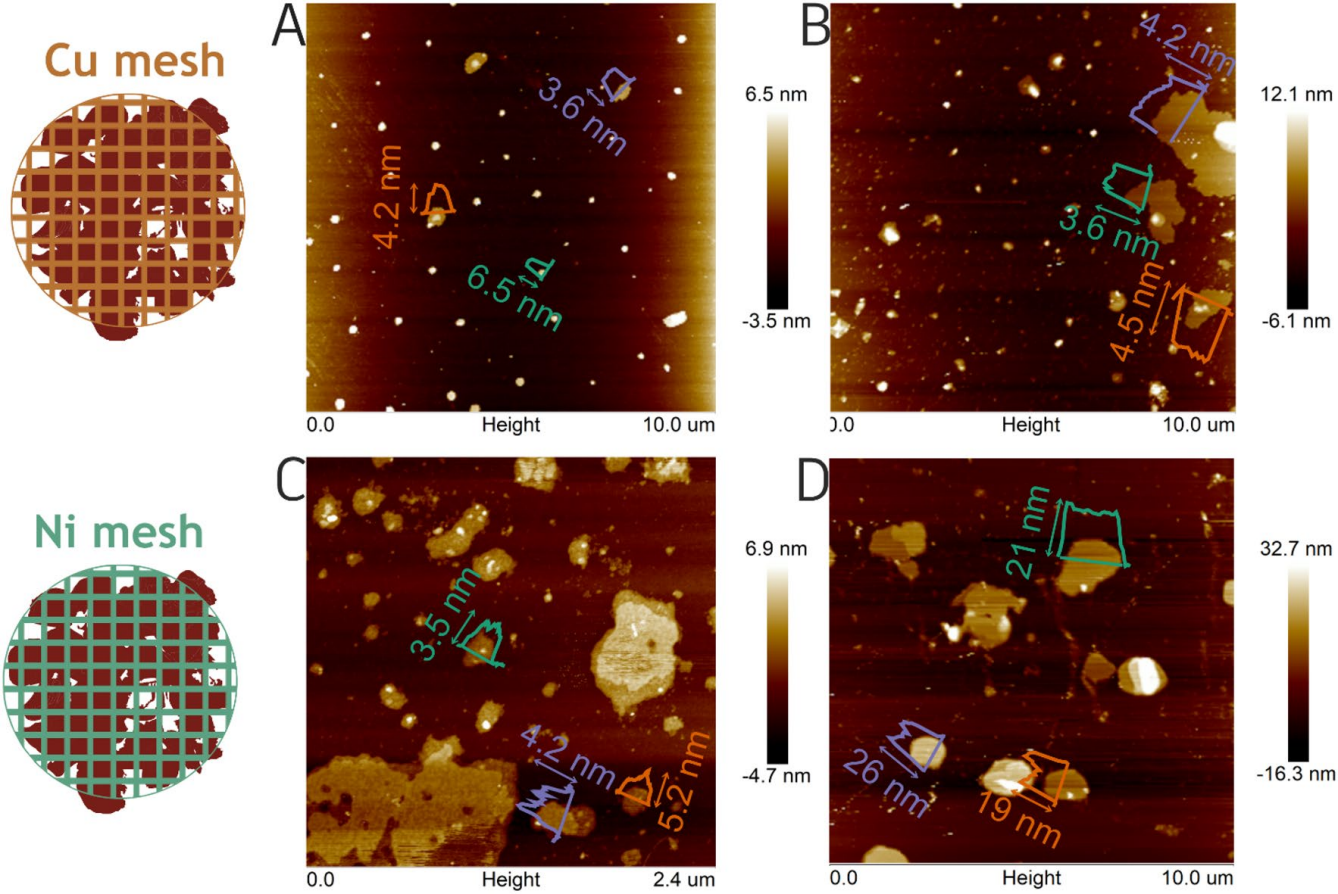
AFM images of borophene sheets after electrochemical exfoliation on **A** Cu_Li^+^_1 A, **B** Cu_SO_4_^2−^_1 A, **C** Ni_Li^+^_1 A and **D** Ni_SO_4_^2−^_1 A.

Initially, the influence of mentioned electrolytes has been revealed at a current of 1 A. The process took 1 h with two types of metal meshes (Ni and Cu), respectively. Figure 2 presents Atomic Force Microscopy (AFM) images of obtained materials and corresponding height profiles are presented in Figure S1. Additionally, the height and size of the sheets fabricated in every experiment were presented in Table 1. Cleary, using Na_2_SO_4_ as an electrolyte the thickness of the flakes is much smaller when Cu mesh is applied. The thickness is reduced ~ 5 times in respect to the flakes exfoliated in the presence of Ni support. Interestingly, the distribution of the size of the flakes is similar. However, LiCl/DMSO was efficient in the exfoliation process using both metal meshes leading to 5–15 layers of borophene, similarly to other liquid-based exfoliations resulting in few-layered sheets of borophene^7,8^. Therefore, in further study detailed structure of the sample exfoliated in this electrolyte will be revealed.

**Table 1:** Summary of height and size ranges after each experiment.

Copper mesh	Nickel mesh				
Sample	Hight (nm)	Size (µm)	Sample	Hight (nm)	Size (µm)
Cu_SO_4_^2−^_1 A	2.71–4.97	0.11–1.47	Ni_SO_4_^2−^_1 A	13.04–26.28	0.53–1.22
Cu_Li^+^_1 A	3.41–6.53	0.19–0.39	Ni_Li^+^_1 A	2.10–5.27	0.16–0.53
Cu_Li^+^_0.5 A	3.12–5.53	0.59–2.67	Ni_Li^+^_0.5 A	4.82–5.27	0.27–1.05
Cu_Li^+^_0.1 A	4.76–5.67	0.78–1.46	Ni_Li^+^_0.1 A	–	–

**Figure 3:**
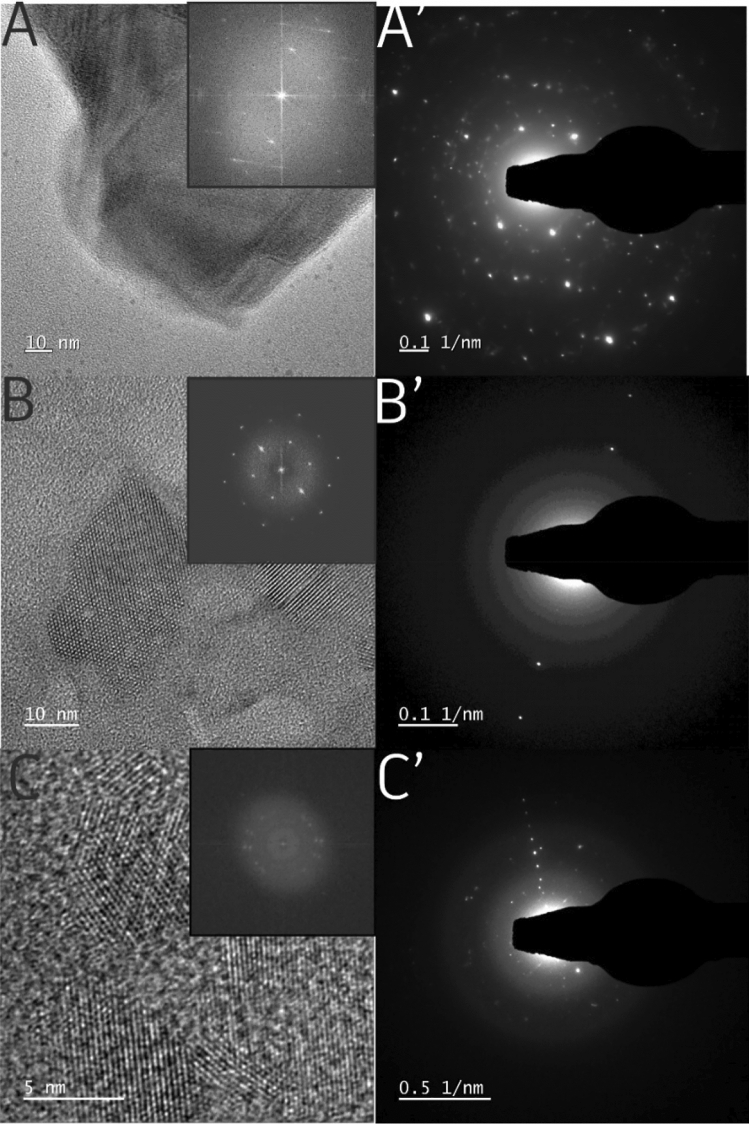
TEM images of **A** bulk boron, **B** Cu_Li^+^_1 A and **C** Ni_Li^+^_1 A with corresponding SAED patterns (**A’**, **B’**, **C’**); insets of fast Fourier transform (FFT) of TEM images.

Transmission Electron Microscopy (TEM) analysis was performed. As shown in Fig. 3 the bulk boron structure is crystalline which is presented in both TEM images of boron and exfoliated boron and corresponding fast Fourier transforms (FFT) followed by Selected Area Electron Diffraction (SAED) patterns. The main difference in the sample after the exfoliation process can be easily noticed in TEM images, where d-spacings are much sharper and the distances are significantly lower (0.35–0.9 nm; Table S2). Although the sample fabricated on Cu-mesh matches the *β-rhombohedral* boron structure^8^, while the one obtained with the use of Ni-mesh is consistent with the theoretical predictions for lattice parameters: β_12_ and χ_3_^17^. It proves that the borophene structure is crystalline but the thickness and crystal structure was changed upon exfoliation. However, it clearly indicates the dependence of the used mesh (Cu or Ni) on the crystallinity of the obtained borophene. It can be a single crystalline or polycrystalline for Cu or Ni, respectively. The crystal modifications were also detected in other exfoliation methods^18,19^. In our case, d-spacing and final structure strongly depend on the sort of applied mesh (Ni, Cu). The considerable change in SAED patterns can be detected suggesting that our method leads to the formation of more homogeneous crystalline structures. Additionally, elemental mapping (EDX) and STEM images prove that manufactured 2D material is composed of boron elements (Figure S5). However, for a deeper understanding of the structure further investigations of the properties of fabricated borophene are required. Especially, the analyses focused on the edges of borophene should be proceeded as they play a crucial role in the stability of the material and its catalytic performance^20–22^.

X-Ray Photoelectron Spectroscopy (XPS) was conducted to determine the oxidation level of the borophene sample. During the heating of the borophene sample, the boron-boron bonding fraction has increased from 6.97 to 28.13% (Table S3). At the same time, the boron-suboxide (B-O) bonding has decreased mainly due to the detachment of surface oxide as well as a transition from boron-suboxide to B_2_O_3_, which is suggested by an increase in the amount of B_2_O_3_ in the sample. The change in bonding ratio during heating is presented in Figure S8 for boron and oxide elements. The overall spectra are presented in Figure S7. The examination shows that the borophene was oxidized on the surface and the boron:oxide ratio before heating was 1:1, and after heating 1.5:1. XPS description in greater detail is provided in the supplementary information.

**Figure 4:**
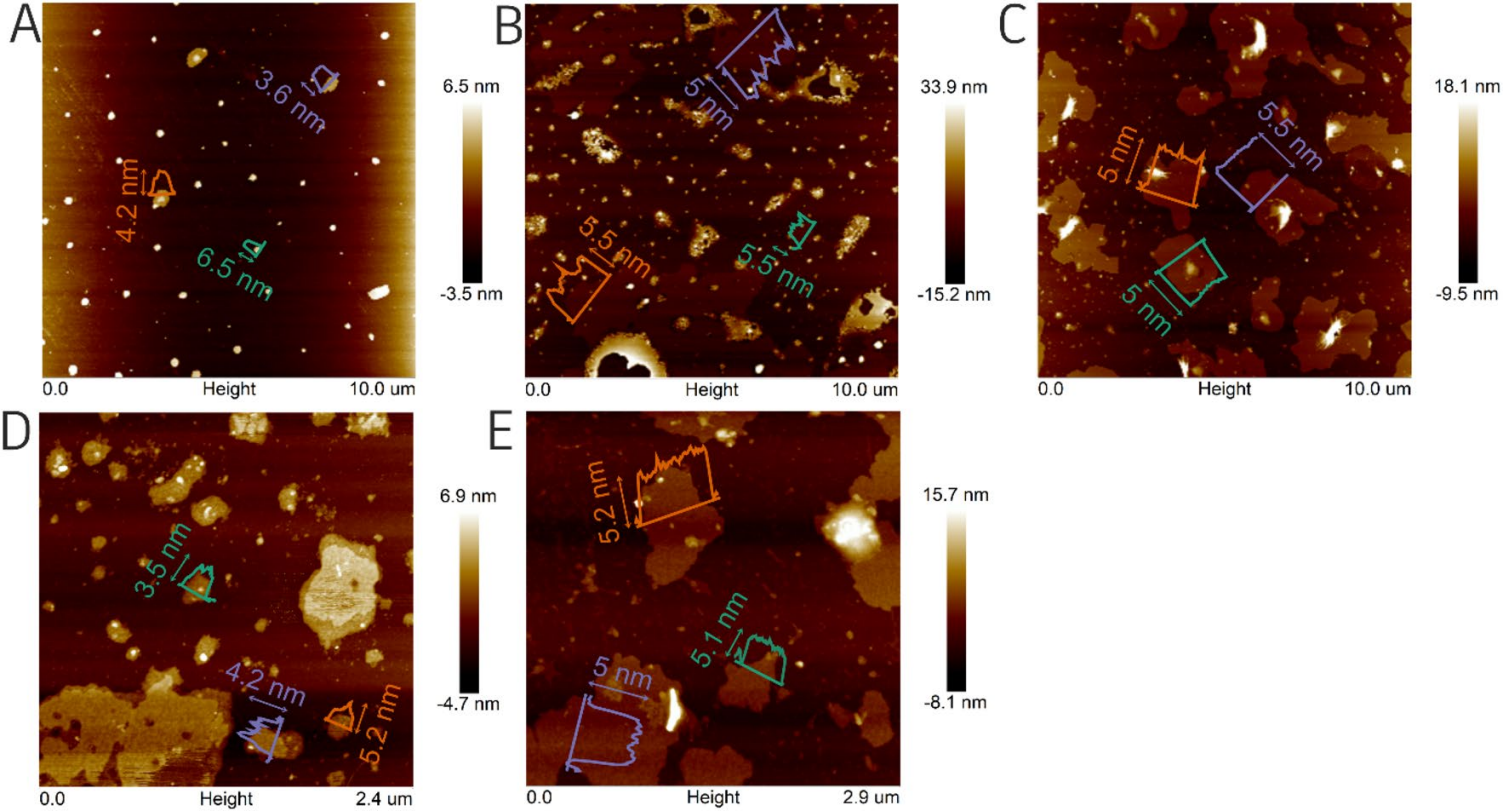
AFM images of boron after electrochemical exfoliation. (**A**) Cu_Li^+^_1 A, (**B**) Cu_Li^+^_0.5 A, (**C**) Cu_Li^+^_0.1 A, (**D**) Ni_Li^+^_1 A, (**E**) Ni_Li^+^_0.5 A.

Next experiments have been conducted to verify the influence of applied current between electrodes during the electrochemical exfoliation. The test has been performed with a current of 0.5 A and 0.1 A in LiCl/DMSO, respectively. The results of the AFM microscopy study are presented in Fig. 4 and corresponding height profiles are in Figures S2 and S3. Considering that a single monolayer of borophene is ~ 0.4 nm thick^12,23^, the thinnest flakes corresponded to 5–11 layers of borophene with lateral size of ~ 0.6–2.5 µm in the experiment at 0.5 A and in the presence of copper mesh. Additionally, the experiment with Ni mesh fabricated the flakes with an extremely small thickness distribution (4.82–5.27 nm). What is interesting, the borophene flakes induced via sonochemical methods yield with similar flake sizes in the range of 1.32–2.32 nm^7^ or 1.8–4.7 nm^8^. Additionally, the electrochemical exfoliation of graphene presented by Achee et al.^14^ resulted in much larger flakes (> 30 µm) what can be due to the size of the starting materials. However, the thickness of the graphene flakes was 2–7 nm. By decreasing the applied current from 1 to 0.1 A more uniform flakes in size and height have been achieved. Therefore, it is a facile strategy to control this crucial textural parameter of 2D material. It is worth noting that the experiment conducted on nickel mesh with a current 0.1 A was not successful. This was caused by the fact that nickel compared to copper possesses a lower electrical conductivity and an insufficient amount of energy was supplied to induce borophene formation^24^. TEM analysis of Cu_Li^+^_0.5 A, Cu_Li^+^_0.1 A, Cu_SO_4_^2-^_1 A, Ni_Li^−^_0.5 A, and Ni_SO_4_^2−^_1 A is presented in Figure S3 and Figure S4 respectively.

**Figure 5:**
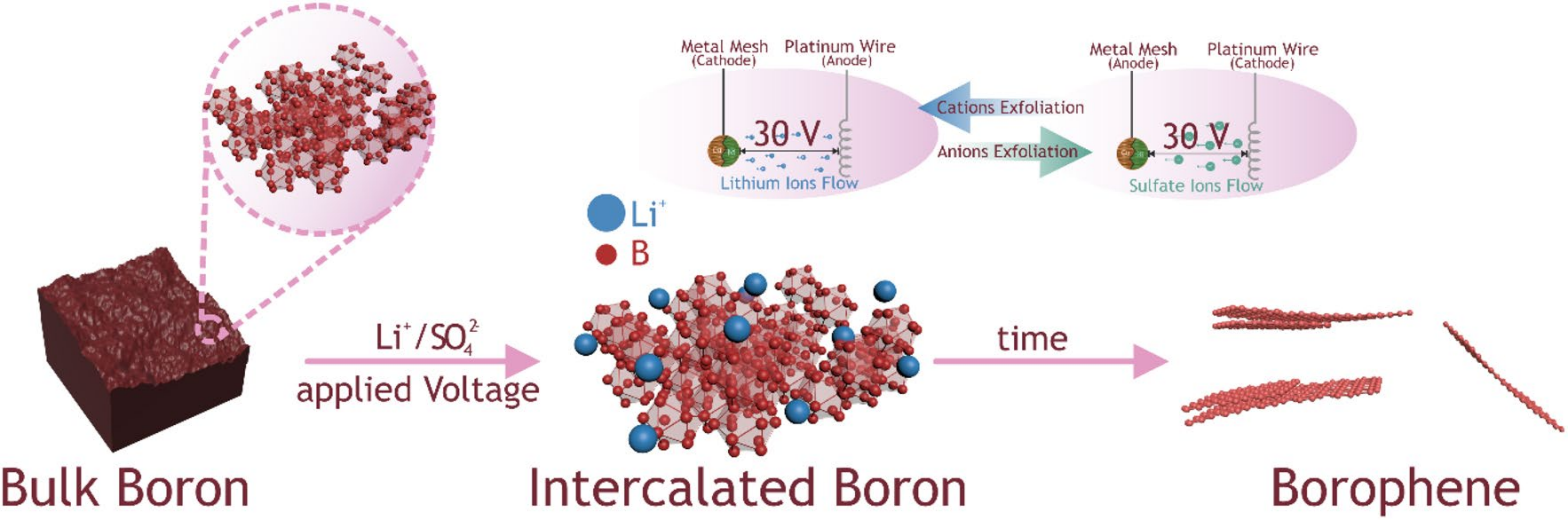
Proposed mechanism of bulk boron exfoliation to few-layered borophene sheets.

Here, we also propose the possible mechanism of bulk boron exfoliation to few-layered borophene (Fig. 5). Initially, the bulk boron is pressed into Cu/Ni mesh to induce electric conductivity of the electrode to successfully apply the voltage between the auxiliary electrode (Pt wire) and the working electrode. That allows ions to migrate through the electrolyte and intercalate the cathode/anode material, depending on the used electrolyte**.** The fact that no ions are released from metal mesh during the process was demonstrated with AAS analysis (see Supplementary Information). It proved that only ions from electrolyte penetrate the boron structure. Bulk commercial boron utilized in this procedure is commonly called “amorphous boron” due to its disordered distribution of primary cell units–*icosahedrons*, *B*_*12*_, which heated to 1000 ℃ forms the ordered *β-rhombohedral* structure (Figure S6)^25^. According to that the lithium cations easily intercalate the boron structure in the first stage and in time rip off the fragments of *B*_*12*_ cells forming 2D structures of borophene with highly ordered structures e.g. *β-rhombohedral*, β_12_ or χ_3_, depending on applied current and mesh material. To reveal the affinity of Li^+^ to bulk boron and its key role in the exfoliation process its Zeta potential (ZP) was measured and it was equal to − 38 ± 3.5 mV (see Supplementary Information). The negative value of ZP of bulk boron indicates that the intercalation by positive lithium cations is more efficient in comparison to other ions used in this study e.g. SO_4_^2−^. This also explains the more efficient penetration of Li^+^ into boron structure yielding more efficient electrochemical exfoliation.

## Conclusion

In summary, we have developed a new fabrication route of few-layered borophene via electrochemical exfoliation of the boron with the utilization of Cu/Ni meshes in Li^+^/DMSO and SO_4_^2−^/H_2_O solutions. It also appears to yield in different phases depending on the applied current and used mesh. The mechanism of the exfoliation process is also proposed and discussed. It can be concluded that by choosing an appropriate metal mesh as support of boron and optimization of applied current it is facile to manufacture quality controlled few-layer borophene which can be further used for fundamental study or practical applications. What is more, it is the first successful attempt at electrochemical boron exfoliation. It is believed that this route can be universal to exfoliate other nonconductive material into their 2D forms. Nevertheless, a deeper understanding of the structure and properties of the synthesized few-layered borophene is still required and more studies should be conducted.

## Supplementary Information

Supplementary Information.

## Data Availability

The datasets generated during and/or analyzed during the current study are available in the RepOD repository, https://doi.org/10.18150/X5LWAN.
