# Supplementary material for: Bottom up approach of metal assisted electrochemical exfoliation of boron towards borophene
Source: Sci Rep. 2022 Sep 20;12:15683. doi: 10.1038/s41598-022-20130-w (PMC9489866; doi:10.1038/s41598-022-20130-w)
Supplement: Supplementary file 1 — Supplementary Information. [file 41598_2022_20130_MOESM1_ESM.docx]

**Electronic Supporting Information**

**Table S1.** Name explanation of the studied samples.

| Sample | Foam | Electrolyte | Current |
| --- | --- | --- | --- |
| Cu_Li^+^_1A | Copper foam | 1M LiCl in DMSO | 1 A |
| Cu_Li^+^_0.5A |  |  | 0.5 A |
| Cu_Li^+^_0.1A |  |  | 0.1 A |
| Cu_SO_4_^2-^_1A |  | 1M Na_2_SO_4_ | 1 A |
| Ni_Li^+^_1A | Nickel foam | 1M LiCl in DMSO | 1 A |
| Ni_Li^+^_0.5A |  |  | 0.5 A |
| Ni_Li^+^_0.1A |  |  | 0.1 A |
| Ni_SO_4_^2-^_1A |  | 1M Na_2_SO_4_ | 1 A |


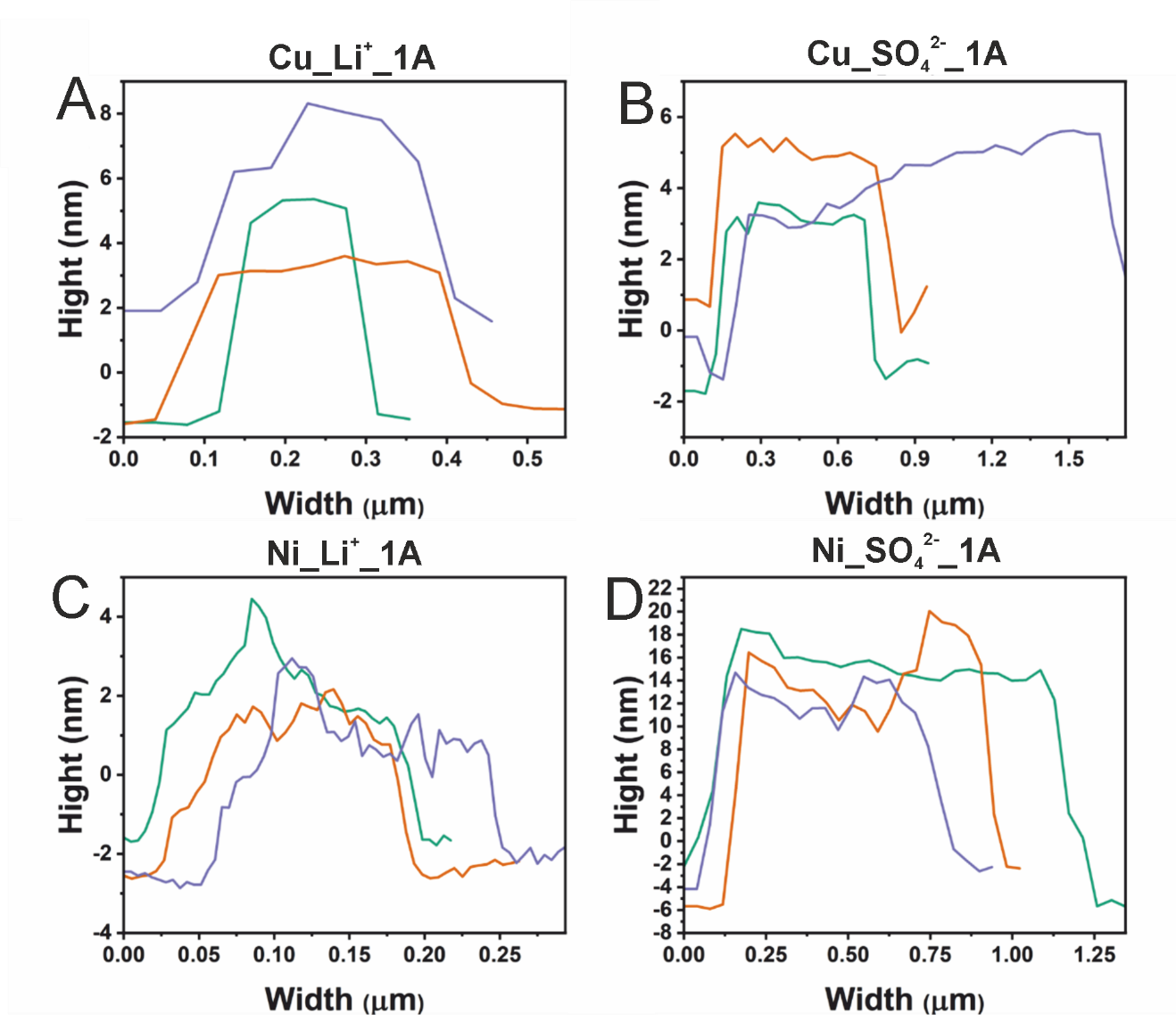


**Figure S1.** Height profiles corresponding to AFM images of exfoliated boron: (A) Cu_Li^+^_1A, (B) Cu_SO_4_^2-^_1A, (C) Ni_Li^+^_1A and (D) Ni_SO_4_^2-^_1A.


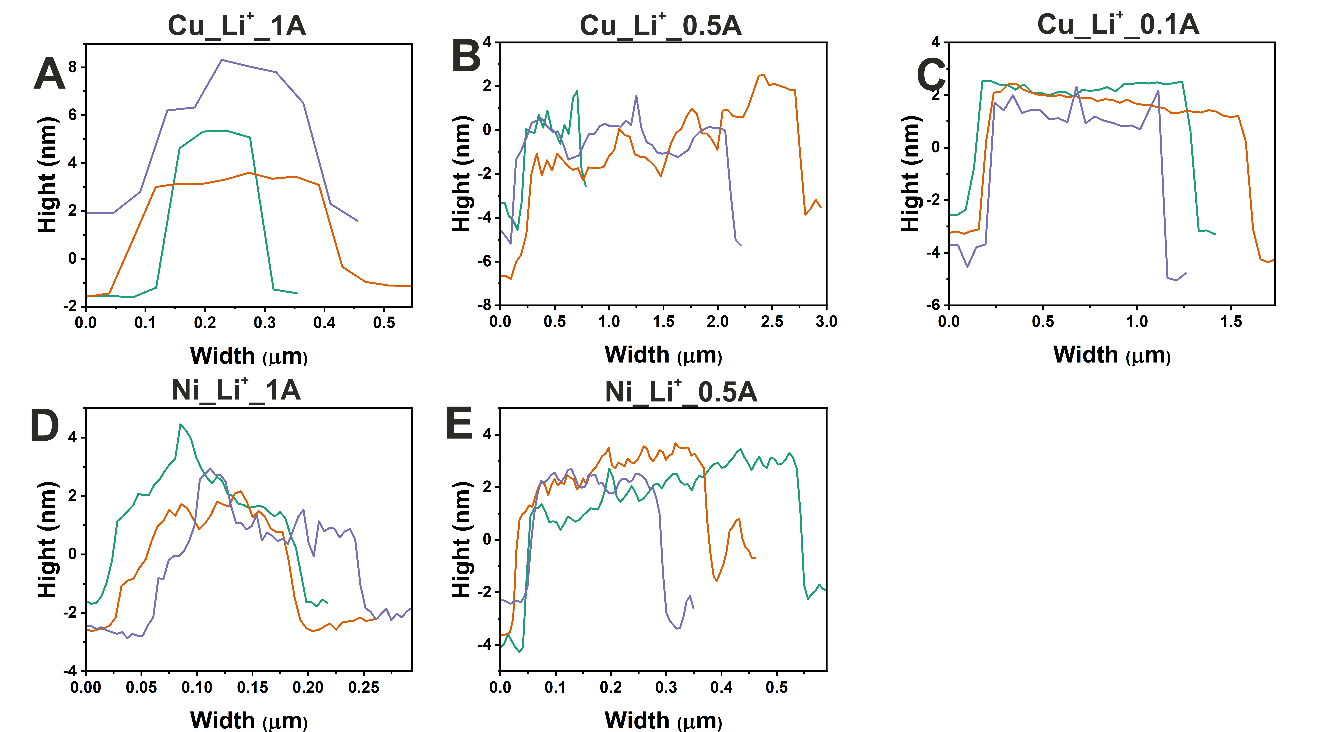


**Figure S2.** Height profiles corresponding to AFM images of exfoliated boron: (A) Cu_Li^+^_1A, (B) Cu_Li^+^_0.5A, (C) Cu_Li^+^_0.1A, (D) Ni_Li^+^_1A, (E) Ni_Li^+^_0.5A.


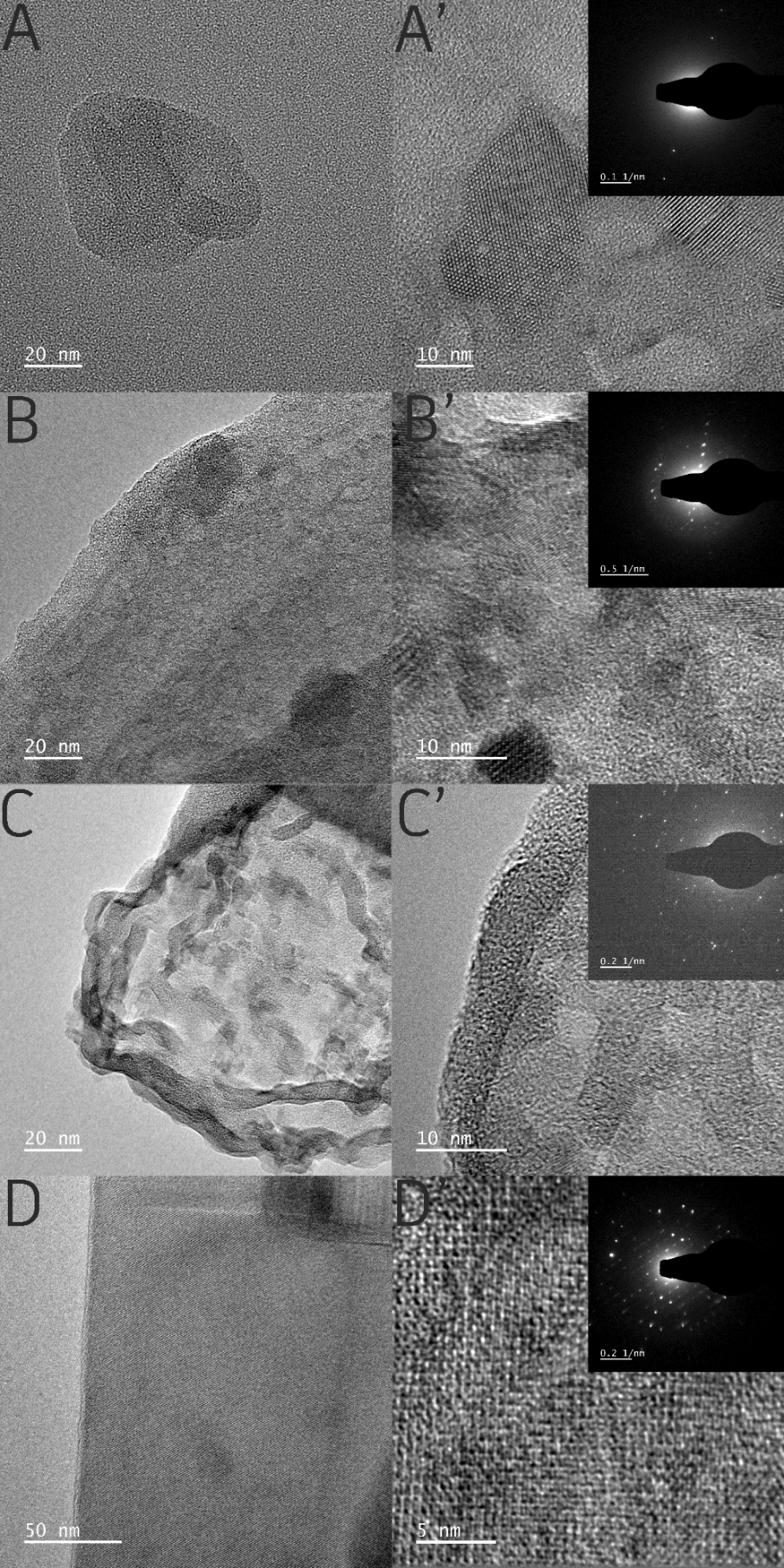


**Figure S3.** TEM images of (A;A’) Cu_Li^+^_1A, (B;B’) Cu_Li^+^_0.5A, (C;C’) Cu_Li^+^_0.1A (D;D’) Cu_SO_4_^2-^_1A and insets of corresponding SAED patterns.


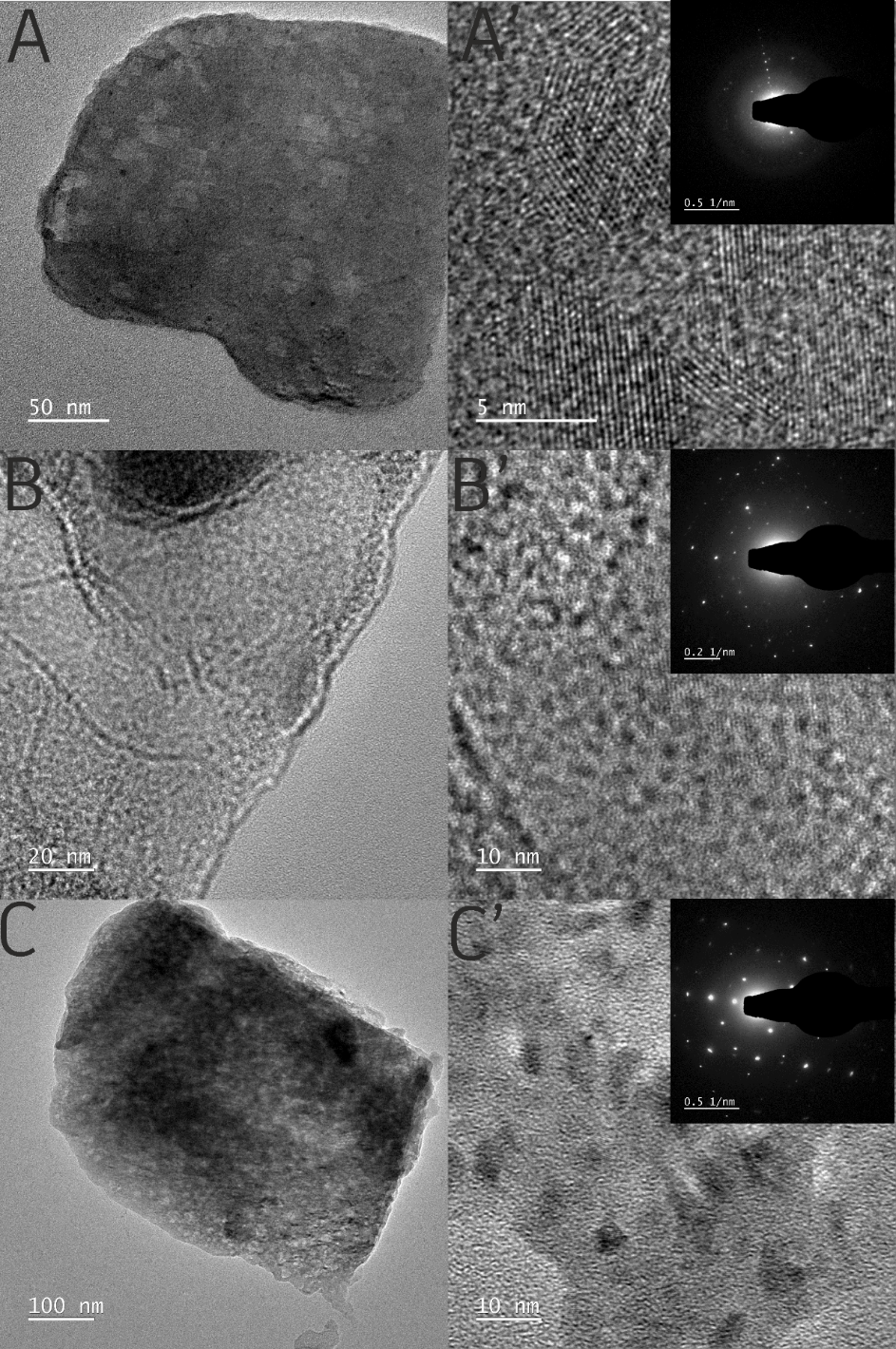


**Figure S4.** TEM images of (A; A’) Ni_Li^+^_1A, (B; B’) Ni_Li^+^_0.5A, (C; C’) Ni_SO_4_^2-_^1A and insets of corresponding SAED patterns.

**Table S2.** The average d-spacing calculated from TEM images for studied borophene materials and bulk boron.

|  | **Ni** | **Cu** | |
| --- | --- | --- | --- |
| **Li^+^_1A** | 0.35 nm | 0.60 nm | |
| **Li^+^_0.5A** | 0.83 nm | 0.75 nm |  |
| **Li^+^_0.1A** | X | 0.50 nm |  |
| **SO_4_^2-^_1A** | 0.42 nm | 0.90 nm |  |
| **BULK BORON** | | 0.41 nm |  |


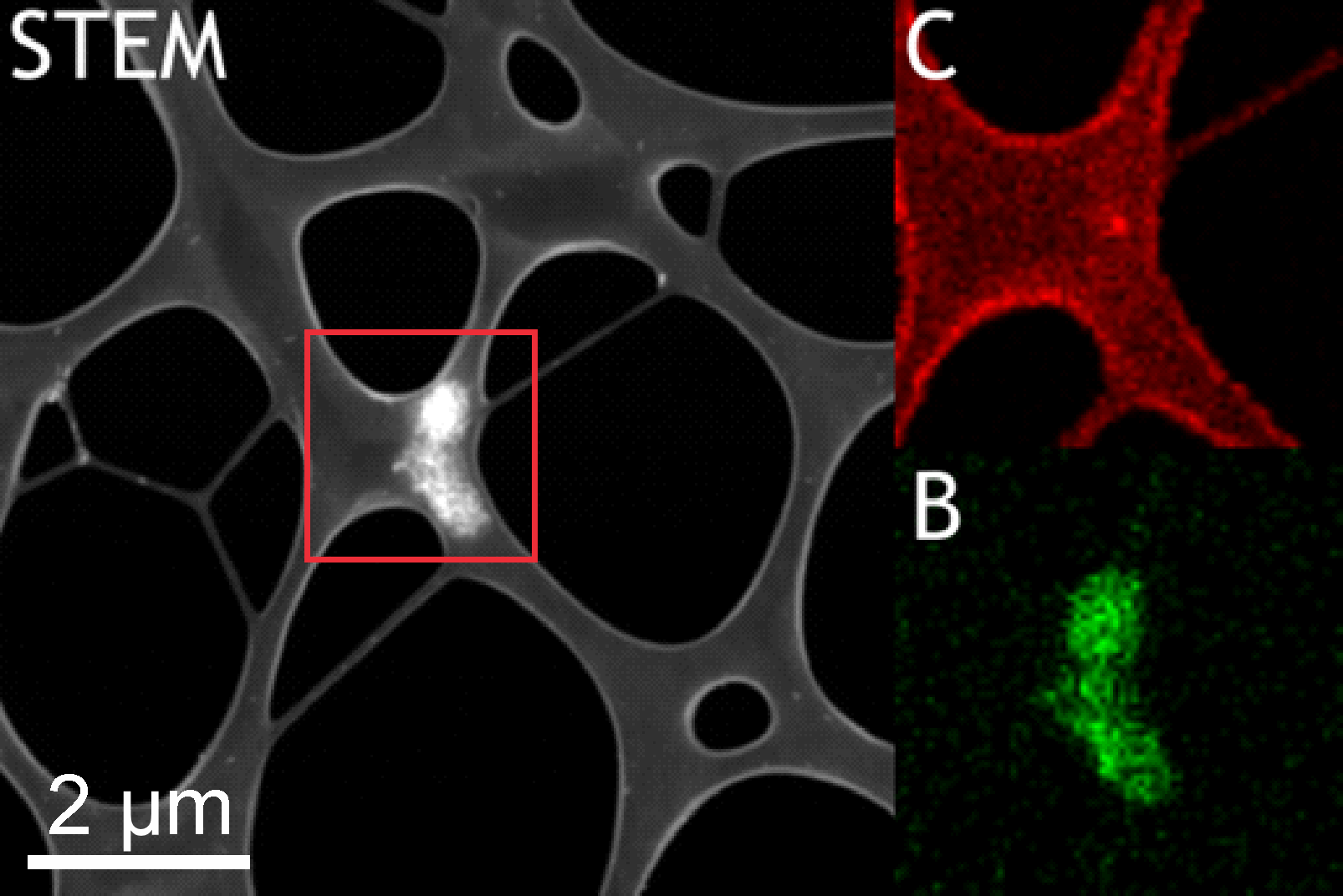


**Figure S5.** STEM image of borophene followed by EDX elemental mapping.

**Figure S6.** XRD patterns of „amorphous” boron and β-rhomboidal boron (after heating amorphous boron in 1000 °C).

*AAS analysis*

The test was engaged to prove that no ions are released from metal mesh during the process which will indicate that no ion is involved in electrochemical exfoliation due to interlayer intercalation. Firstly, six standard solutions with different concentrations were prepared to start from SPEX CentriPrep standard solution of nickel. Each solution with a different concentration was measured three times to obtain a calibration curve. Next, the electrolyte solution (LiCl in DMSO after the experiment performed on nickel mesh with 1 A) was measured without dilution. The test was carried out with a replication of ten times. The results indicate no presence of nickel ions in the electrolyte solution which confirmed the hypothesis that no metal ions from metallic mesh are involved in the electrochemical exfoliation mechanism.

*Zeta potential measurements*

Firstly, the uniform suspension of bulk boron in distilled water was prepared with a concentration of 1 mg/mL. The measurement was conducted with triple replication. The obtained value of zeta potential for boron was -38 ± 3.5 mV.

*X-Ray Photoelectron Spectroscopy*

To determine the oxidation level for our sample we conducted a series of tests in UHV on XPS. Firstly, the sample was prepared by drop-casting of ethanol-borophene solution on graphite foil. This step was necessary due to the need for a not-powdered form of examined sample, as well as an insufficient amount of sample to prepare the tablet. Firstly, the prepared sample was analyzed at room temperature (RT). After analysis, the sample was transferred inside the XPS instrument to the reactor, where was heated to 200°C for 2 hours with hydrogen flow (30 mL/min). After this process, the sample was immediately transferred to analysis. The same conditions were maintained for heating to 400°C. The goal of this process was to evaluate the change in the boron and oxygen ratio. As it can be seen the total boron content in the sample was increased from (17.6 at.% to 30.5 at.%) (please see Table 1.). Also, from deconvoluted boron spectra, it can be noticed that the boron-boron bonding fraction has increased from 6.97% to 28.13%. At the same time, the boron-suboxide (B-O) bonding has decreased mainly due to the detachment of surface oxide as well as a transition from boron-suboxide to B_2_O_3_, which is suggested by increasing the amount of B_2_O_3_ in the sample. Also, altering the heating process the nitrogen and fluorine were almost completely detached from the surface. Both, N and F atoms are present in the sample due to contamination of as purchased bulk boron and are also present in XPS spectra of bulk boron. Nevertheless, this examination shows that the borophene was oxidized on the surface and the boron:oxide ratio before heating was 1:1, and after heating 1.5:1. However, it has to be pointed out that the exact quantitative ratio can not be specified due to: (1) carbon signal from graphite foil which distorts composition quantification, and (2) the presence of different boron derivatives, such as boron-suboxide and B_2_O_3_ formed during the electrochemical process, causing the higher content of oxygen-boron bond. Nonetheless, the removal of oxygen from the surface and stabilization of obtained borophene are interesting aspects for future investigation.


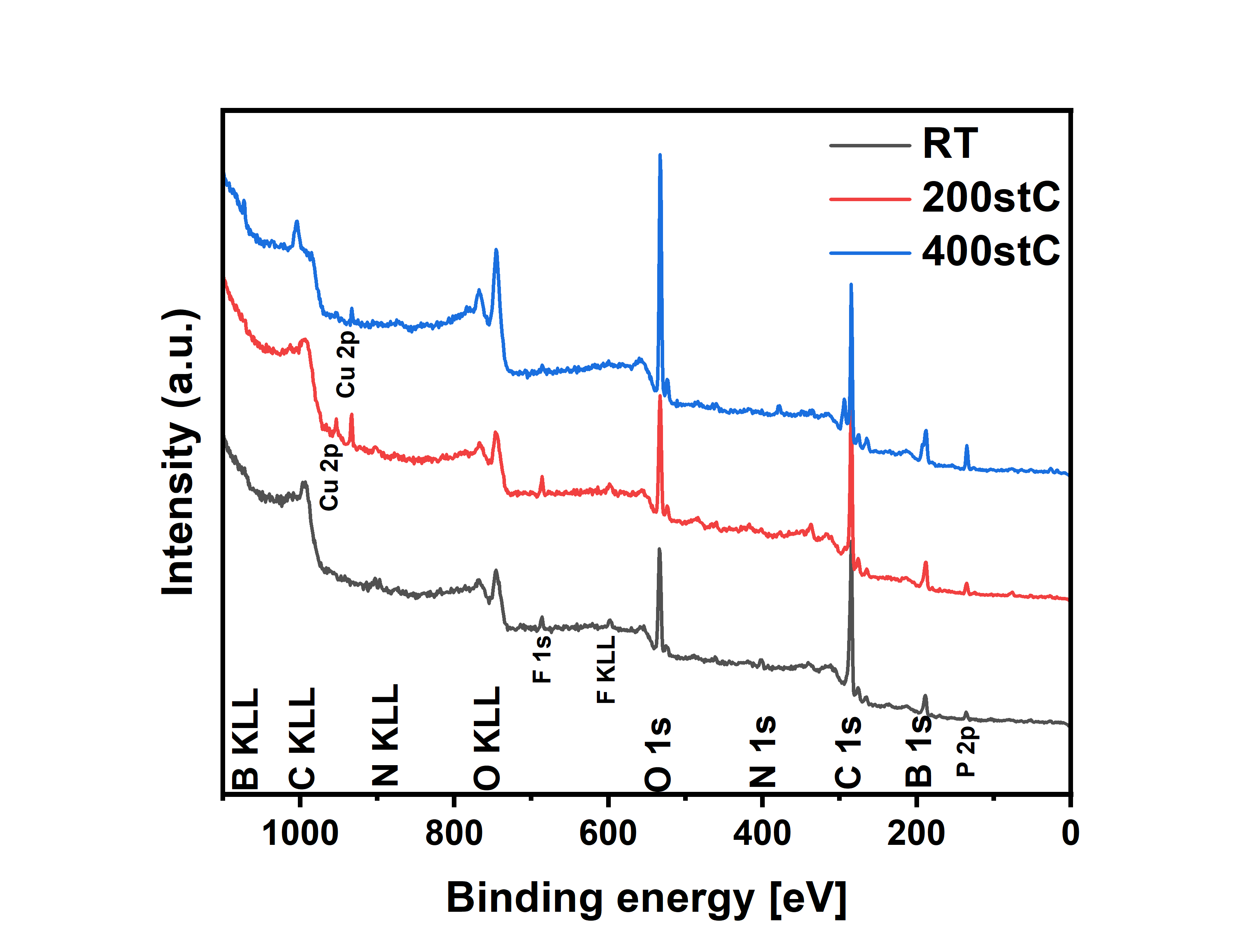


**Figure S7.** XPS survey spectra of borophene without heating (grey line), with heating up to 200°C with hydrogen flow (30 mL/min) (red line) and with heating up to 400°C with hydrogen flow (30 mL/min) (blue line). The heating process takes place inside the UHV XPS system in the reactor.


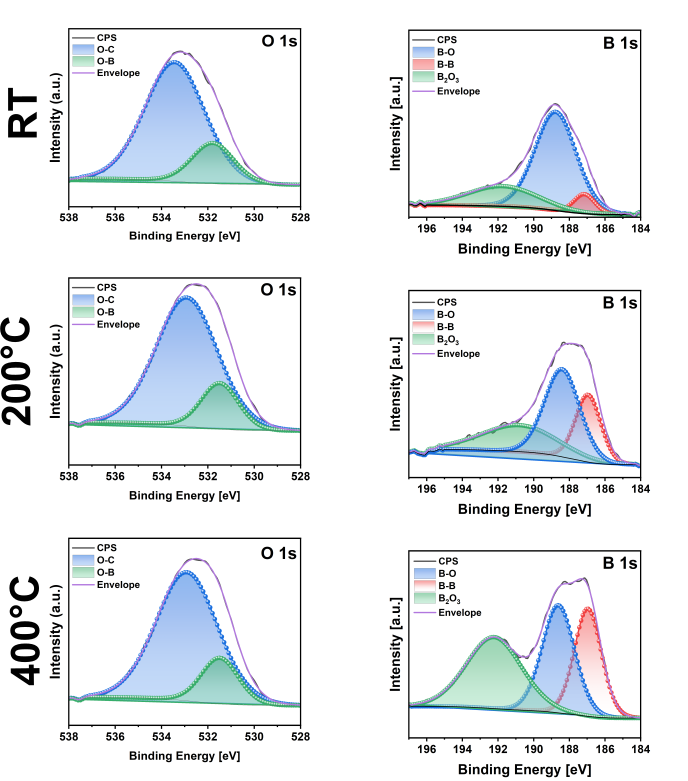


**Figure S8.** XPS deconvoluted spectra of oxygen and boron obtained before heating (top), after heating to 200°C (middle) and after 400°C (down).

**Table S3.** XPS data of composition from survey spectra for borophene samples heated in different temperatures and bond fraction for carbon and oxygen after deconvolution.

| Temperature | Composition (at.%) | | | | | Bond Fraction in O1s(%) | | Bond Fraction in B1s(%) | | |
| --- | --- | --- | --- | --- | --- | --- | --- | --- | --- | --- |
|  | **B** | **C** | **N** | **F** | **O** | **O-B** | **O-C** | **B-B** | **B-O** | **B_2_O_3_** |
| RT | 17.6 | 62.6 | 1.9 | 1.4 | 16.6 | 19.34 | 80.66 | 6.97 | 69.35 | 23.68 |
| 200°C | 24.6 | 58.4 | 0.9 | 1.2 | 14.8 | 17.81 | 82.19 | 25.11 | 45.61 | 29.28 |
| 400°C | 30.5 | 47.4 | 0.0 | 0.3 | 21.8 | 51.4 | 48.6 | 28.13 | 33.72 | 38.15 |


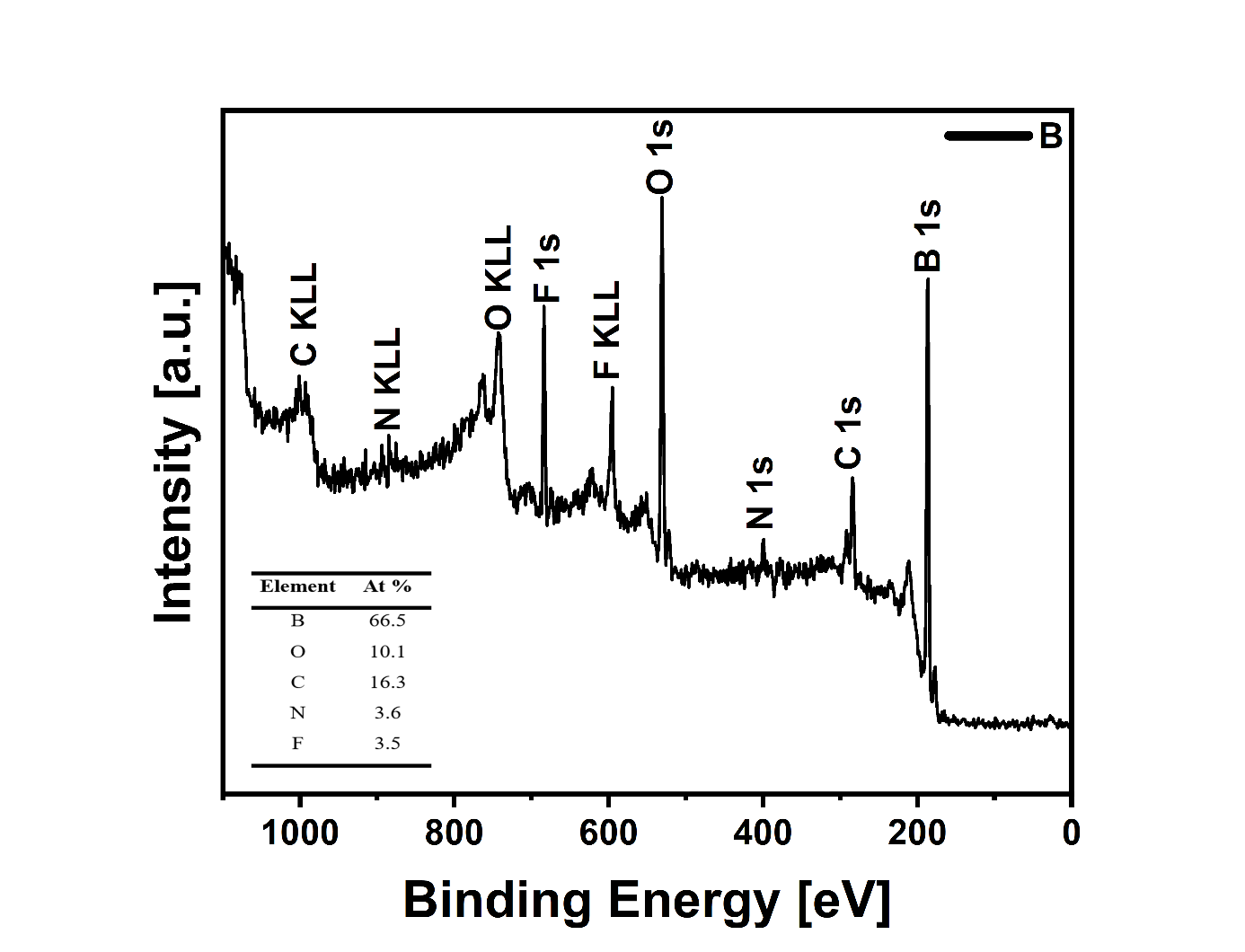


**Figure S9.** XPS survey spectra of as-purchased boron
